# Supplementary material for: SOM-LWL method for identification of COVID-19 on chest X-rays
Source: PLoS One. 2021 Feb 24;16(2):e0247176. doi: 10.1371/journal.pone.0247176 (PMC7904146; doi:10.1371/journal.pone.0247176)
Supplement: Attachment — Submitted filename: Response to Reviewers.doc [file pone.0247176.s001.doc]

**Original Manuscript ID:** PONE-D-20-32000

**Original Article Title: “**SOM-LWL Method for Identification of COVID-19 on Chest X-rays”

**To:** PLOS ONE Editor

**Re:** Response to reviewers

Dear Editor,

Thank you for allowing a resubmission of our manuscript, with an opportunity to address the reviewers’ comments.

We are uploading (a) our point-by-point response to the comments (below) (response to reviewers), (b) an updated manuscript with yellow highlighting indicating changes, and (c) a clean updated manuscript without highlights (PDF main document).

Best regards,

Ahmed Hamza Osman

Corresponding Author

Thank you very much for your valuable comments. We have revised the manuscript according to your comments. Your comments help us improve the quality of the paper a lot. Thank you again!

**Concern # 1: characteristics of included patients are not clearly provided.**

**Author response:** *The* *characteristics of included patients are updated and reported and now clearer. Please see Section V – dataset subsection B.*

**Concern # 2: Analysis of recent related papers is missing**

***Author response:*** *The analysis of recent related papers has been reviewed and reported to be more solid. In addition, the manuscript* *has been revised accordingly. Please see Section II – Related works.*

**Concern # 3: figures 1, 5 and 11 are not clear,**

***Author response:*** *The figures 1, 5 and 11 has been redrawn to be clearer. Please see figures 1, 5 and 11 in the paper.*

 **Concern # 4: it is not clear if cross-validation scheme was used**

***Author response:*** *The Cross validation scheme has been used and involved in our experiments. The simple idea of holdout by using certain information for testing and the rest for training. In 10-fold cross validation, the contents of one fold are influenced by the contents of other folds. The different cross-validations are applied with independent samples from the COVID-19 dataset to obtain some variations in results and remove any outliers based on averaging. The COVID-19 dataset was divided into 10 folds after the data balancing process. Two different scenarios have been used for the identification and classification of COVID-19 in X-rays. First, the SOM-LWL scheme is trained to classify the X-rays into three classes: COVID-19, Non-COVID-19, and pneumonia. Furthermore, two classes are trained with the SOM-LWL model: the COVID-19 classes and the Non-COVID-19 classes. For triple and binary classification problems, the output of the suggested model is assessed by the 10-fold cross validation process. The training records use 90% of the X-ray images and 10% as the testing stage, the process is performed four times based on the balance of the dataset that has been determined in pre-processing phase. In contrast to the traditional hold-out validation process, this type of validation method provides better results.*

*Please see Section V – subsection A.*

**Concern # 5: Proofreading is required to fix typos.**

Author response: *The English of the paper was revised and the paper was sent to the American Journal Experts (AJE) proofreading services to fix all the typos and the Grammar errors in the paper as well. A proofreading certificate was provided and attached to prove that.*

Finally, we are very thankful to the anonymous reviewers for his very useful suggestions and comments. I hope the paper could have made an improvement this time.

**Yours sincerely**

**Ahmed Hamza Osman**

Corresponding Author
