# Supplementary material for: SOM-LWL method for identification of COVID-19 on chest X-rays
Source: PLoS One. 2021 Feb 24;16(2):e0247176. doi: 10.1371/journal.pone.0247176 (PMC7904146; doi:10.1371/journal.pone.0247176)
Supplement: Attachment — Submitted filename: Response to Reviewers.doc [file pone.0247176.s002.doc]

**Original Manuscript ID:** PONE-D-20-32000R1

**Original Article Title: “**SOM-LWL Method for Identification of COVID-19 on Chest X-rays”

**To:** PLOS ONE Editor

**Re:** Response to reviewers

Dear Editor,

Thank you for allowing a resubmission of our manuscript, with an opportunity to address the reviewers’ comments.

We are uploading (a) our point-by-point response to the comments (below) (response to reviewers), (b) an updated manuscript with yellow highlighting indicating changes, and (c) a clean updated manuscript without highlights (PDF main document).

Best regards,

Ahmed Hamza Osman

Corresponding Author

**Reviewer # 1**

All comments have been addressed

**Thank you very much for your valuable comments. We have revised the manuscript according to your comments. Your comments help us improve the quality of the paper a lot. Thank you again!**

**Reviewer # 2**

**First we apologize for not including your comments in the previous round, not intentionally, but by mistake. Certainly, your comments are of interest to us and have greatly improved the form of our manuscript in terms of knowledge and representation. Thank you very much for your valuable comments. We hope that included all your comments this time in a way that meets your comments.**

**This paper presented a new COVID-19 identification method based on the locality-weighted learning and self-organization map (LWL-SOM) strategy for detecting and capturing COVID-19 cases. The topic is interesting, I congrats the authors for your effort. However, some aspects should be improved.**

**Concern # 1: The abstract can be rewritten to be more meaningful. The authors should add more details about their final results in the abstract. Abstract should clarify what is exactly proposed (the technical contribution) and how the proposed approach is validated.**

**Author response:**

*The abstract has been revised and the finding results has been reported and highlighted. In addition the technical contributions are updated and now clearer. Please see the abstract of the paper.*

**Concern # 2: Bullet your contribution at the end of the introduction section.**

**Author response:** *The contributions of the* *proposed method has been updated and highlighted in the abstract, and the end of the introduction section. Please see the introduction section.*

**Concern # 3: Many recent papers are missing such as:**

**Deploying machine and deep learning models for efficient data-augmented detection of covid-19 infections. Viruses 12, no. 7 (2020): 769.**

**A Deep Learning Approach to Detect COVID-19 Patients from Chest X-ray Images. AI 1, no. 3 (2020): 418-435.**

**A Survey on Artificial Intelligence Approaches in Supporting Frontline Workers and Decision Makers for COVID-19 Pandemic. Chaos, Solitons & Fractals (2020): 110337.**

***Author response:*** *The mentioned of recent related papers and other papers has been reviewed and reported to be more solid. In addition, the manuscript* *has been revised accordingly. Please see Section 2 – Related works.*

**Concern # 4: Figures 1, 5 and 11 not clear, I would ask author to redesign this figure with clear details.**

***Author response:*** *The figures 1, 5 and 11 has been redrawn to be clearer. Please see figures 1, 5 and 11 in the paper.*

 **Concern # 5: I recommend adding a Table for related works and show the advantages and disadvantages of each study.**

***Author response:*** *The advantages and disadvantages of related works has been investigated and highlighted in the form of table as the reviewer suggested. Please see the end of related work section.*

**Concern # 6: Authors need to provide justifications for all the parameters setting.**

***Author response****: all the parameters setting has been justified and provided in the paper in details. Please see the section III proposed method section.*

**Concern # 7: Overall, the manuscript has potential, if more dataset can be including in the analysis will be beneficial for reaching a concrete conclusion such as:**

- **Combined COVID-19 Dataset, 2020, Mendeley Data, V3, doi: 10.17632/3pxjb8knp7.3.**
- **Noisy COVID-19 X-ray Dataset, 2020, Mendeley Data, V3, doi: 10.17632/fjg5cbzffh.3**

***Author response****: The mentioned datasets are focused on the CT images combined with X-ray images. In addition, the number of confirmed, recovered, and death in specific date and country while the proposed method focused on extracting the features of X-ray images only from both COVID-19 and Non-COVID-19 patients. We think these datasets will be suitable for regression model rather than classification model. Furthermore, we declared these datasets in the Conclusion and Future work section to be one of our priorities and the priorities of researchers in our next paper. Please see the section VII Conclusion and Future work section.*

**Concern # 8: Please highlight the advantages and disadvantages of your method.**

***Author response****: The advantages and limitations of the proposed method has been provided and highlighted in the introduction section.*

**Concern # 9: it is not clear if cross-validation scheme was used**

***Author response:*** *The Cross validation scheme has been used and involved in our experiments. The simple idea of holdout by using certain information for testing and the rest for training. In 10-fold cross validation, the contents of one fold are influenced by the contents of other folds. The different cross-validations are applied with independent samples from the COVID-19 dataset to obtain some variations in results and remove any outliers based on averaging. The COVID-19 dataset was divided into 10 folds after the data balancing process. Two different scenarios have been used for the identification and classification of COVID-19 in X-rays. First, the SOM-LWL scheme is trained to classify the X-rays into three classes: COVID-19, Non-COVID-19, and pneumonia. Furthermore, two classes are trained with the SOM-LWL model: the COVID-19 classes and the Non-COVID-19 classes. For triple and binary classification problems, the output of the suggested model is assessed by the 10-fold cross validation process. The training records use 90% of the X-ray images and 10% as the testing stage, the process is performed four times based on the balance of the dataset that has been determined in pre-processing phase. In contrast to the traditional hold-out validation process, this type of validation method provides better results.*

*Please see Section V – subsection A.*

**Concern # 10: I recommend defining all parameters in a Table at the end of Introduction Section.**

***Author response****: all the parameters setting has been justified and provided in the paper in details. Please see the section III proposed method section.*

**Concern # 5:** • **Whereas overall English quality is good, a thorough proofreading is required to fix some typos.**

Author response: *The English of the paper was revised and the paper was sent to the American Journal Experts (AJE) proofreading services to fix all the typos and the Grammar errors in the paper as well. A proofreading certificate was provided and attached to prove that.*

Finally, we are very thankful to the anonymous reviewers for his very useful suggestions and comments. I hope the paper could have made an improvement this time.

**Yours sincerely**

**Ahmed Hamza Osman**

Corresponding Author
