# Supplementary material for: SOM-LWL method for identification of COVID-19 on chest X-rays
Source: PLoS One. 2021 Feb 24;16(2):e0247176. doi: 10.1371/journal.pone.0247176 (PMC7904146; doi:10.1371/journal.pone.0247176)
Supplement: Attachment — Submitted filename: Response to Reviewers.doc [file pone.0247176.s003.doc]

**Original Manuscript ID:** PONE-D-20-32000R2	(**Minor revision**)

**Original Article Title: “**SOM-LWL Method for Identification of COVID-19 on Chest X-rays”

**To:** PLOS ONE Editor

**Re:** Response to reviewers

Dear Editor,

Thank you for allowing a resubmission of our manuscript, with an opportunity to address the reviewers’ comments.

We are uploading (a) our point-by-point response to the comments (below) (response to reviewers), (b) an updated manuscript with yellow highlighting indicating changes, and (c) a clean updated manuscript without highlights.

Best regards,

Ahmed Hamza Osman

Corresponding Author

**Reviewer # 1**

**I have no competing interests. The authors have responded appropriately.**

**Thank you very much for your valuable comments. We have revised the manuscript according to your comments. Your comments help us improve the quality of the paper a lot. Thank you again!**

**Reviewer # 2**

**The authors have addressed almost of reviewer's concerns and the revised version of the manuscript appears to be good.**

**Minor revision is needed:**

**Concern # 1: Please bullet your contribution at the end of the introduction Section.**

**Author response:**

*The paper contribution was added at the end of the introduction section to be clearer according to the reviewer comment. Please see the end of the introduction section in the manuscript.*

**Concern # 2: Table I, please add the used methods and the performance for each reference.**

**Author response:** Table I was revised according to the reviewer comments by adding the used methods/techniques and its performances. Please see Table I in the manuscript.

**Concern # 3: Add references for your future work (e.g. give references for the data).**

***Author response:*** *The future work section has been revised by adding some references that will be improved this research in future. Please see the future work section.*

**Concern # 4:** **Abstract, still need to add more details about your final results.**

***Author response:*** *The abstract has been revised and the finding of final results has been reported and highlighted too. Please see the abstract of the paper.*

Finally, we are very thankful to the anonymous reviewers for his very useful suggestions and comments. I hope the paper could have made an improvement this time.

**Yours sincerely**

**Ahmed Hamza Osman**

Corresponding Author
